# Knotify+: Toward the Prediction of RNA H-Type Pseudoknots, Including Bulges and Internal Loops

**DOI:** 10.3390/biom13020308

**Published:** 2023-02-06

**Authors:** Evangelos Makris, Angelos Kolaitis, Christos Andrikos, Vrettos Moulos, Panayiotis Tsanakas, Christos Pavlatos

**Affiliations:** 1 School of Electrical and Computer Engineering, National Technical University of Athens, 9 Iroon Polytechniou St., 15780 Athens, Greece; 2 Hellenic Air Force Academy, Dekelia Air Base, Acharnes, 13671 Athens, Greece

**Keywords:** H-type pseudoknot structure, RNA, bulges, internal loops, parser, CFG

## Abstract

The accurate “base pairing” in RNA molecules, which leads to the prediction of RNA secondary structures, is crucial in order to explain unknown biological operations. Recently, COVID-19, a widespread disease, has caused many deaths, affecting humanity in an unprecedented way. SARS-CoV-2, a single-stranded RNA virus, has shown the significance of analyzing these molecules and their structures. This paper aims to create a pioneering framework in the direction of predicting specific RNA structures, leveraging syntactic pattern recognition. The proposed framework, Knotify+, addresses the problem of predicting H-type pseudoknots, including bulges and internal loops, by featuring the power of context-free grammar (CFG). We combine the grammar’s advantages with maximum base pairing and minimum free energy to tackle this ambiguous task in a performant way. Specifically, our proposed methodology, Knotify+, outperforms state-of-the-art frameworks with regards to its accuracy in core stems prediction. Additionally, it performs more accurately in small sequences and presents a comparable accuracy rate in larger ones, while it requires a smaller execution time compared to well-known platforms. The Knotify+ source code and implementation details are available as a public repository on GitHub.

## 1. Introduction

RNA and its functions play a significant role in a variety of biological operations. DNA molecules, where the genetic information is stored, are transcribed into mRNA, which carries the information into the cytoplasm, where translation takes place and leads to the production of a protein. Due to its utmost importance, this procedure is also called the “central dogma” of molecular biology [1]. Apart from that major functionality, RNA has been proven to be involved in a wide range of central biological phenomena, such as gene expression regulation, site recognition, and catalysis [2,3]. All these RNAs, except the mRNA, are called noncoding because they fulfill functions other than encoding proteins, also elaborating the necessity of the detailed analysis of these molecules. In this context, it is vital to predict the structure of RNA, specifically its 3D structure, to understand its functions. This tertiary structure can be determined using techniques such as X-ray crystallography [4] and nuclear magnetic resonance [5]. However, researchers have focused on the development of a methodology toward the prediction of a simpler representation of an RNA structure in a two-dimensional space, named a secondary structure, which is a collection of A (Adenine), U (Uracil), G (Guanine), and C (Cytosine) bases that form duplex regions and unpaired ones that form important motifs around them, such as loops, bulges, and hairpins. Therefore, the secondary structure is this collection of pairs (A–U, C–G, and G–U pairs) that form different motifs. The accurate location of the base pairs and motifs is a useful milestone and starting point for the enlightenment of the 3D structure and, consequently, the understanding of RNA operations.

Recent RNA secondary structure prediction methods have been based mainly on a scoring function that may rely on a thermodynamic, probabilistic, or Artificial Intelligence (AI)-based algorithm. The majority of the methods have utilized or adopted a partially minimum free energy algorithm introduced by Zuker, which facilitates dynamic programming enhanced with parameters from experiments [6]. The Nussinov algorithm is also a widely used method that has succeeded in predicting the largest number of base pairings using dynamic programming [7], which performed even better when it was combined or incorporated as an internal component in other more sophisticated algorithms, as in [8]. Other current approaches have leveraged stochastic methods, syntactic pattern recognition, machine learning, statistical techniques, integer programming, or other heuristic algorithms to tackle the prediction task. Section 2 contains a detailed analysis of the related literature.

In an RNA secondary structure, the pseudoknot’s prediction is the most demanding task in terms of prediction. Other common motifs are stems, hairpins, bulges, internal loops, and multibranch loops, which a variety of algorithms are able to predict with high accuracy. On the other hand, the prediction of a pseudoknot is quite complicated because dynamic programming and minimum free energy algorithms are not constructed in such a way as to facilitate the interconnection of a pseudoknot. Another important reason is that with the increase in the length of the RNA, these algorithms need an exponential execution time. Thus, the need to achieve an accurate prediction for pseudoknots led our research towards constructing a platform that predicts H-type pseudoknots combined with bulges and internal loops with an accuracy similar to well-known methods and, at the same time, an efficiency in terms of execution time, called Knotify+. The H-type pseudoknot [9] consists of two stems and two loops of arbitrary lengths.

Bulge loops or bulges form when a helix is interrupted by unpaired nucleotides on one strand, and they are frequently observed in the secondary structures of RNA [10,11], as they appear in a universal distribution in all types of structured functional RNAs [12]. Base–base mismatches, shaping internal loops, also appear often in RNA, affecting the stability of the molecule [10]. Specifically, researchers have focused on the study of bulges, due to the frequency with which bulged adenosine residues occur at protein binding sites in RNA [10,13], while they also operate as contact points in the tertiary folding of RNA [11,14]. Bulges construct unique recognition sites in RNA tertiary structures in two ways, first by acting as molecular handles within the helical regions and second, in an indirect way, by distorting the RNA backbone and allowing access to base pairs in a widened deep groove [12]. Additionally, helical elements separated by bulges frequently undergo transitions between unstacked and coaxially stacked conformations during the folding and function of noncoding RNAs [12]. All the above references show the importance of the identification of bulges and internal loops as key structural elements in a wide range of RNAs and emphasize their significance and pluralism in RNA architecture and molecular recognition.

In this work, we suggest a new version of Knotify [15], which is capable of predicting bulges and internal loops [12] in an H-type pseudoknot. The sequence of the RNA is imported to a parser which produces the entire set of the possible core stems of a pseudoknot. Next, all these trees are decorated with possible base pairs close to the two stems (core stems) that form the pseudoknot, with the difference that the algorithm is searching for possible bulges and internal loops around it. Towards the prediction of the optimal tree, a set of candidates is created according to the greatest number of base pairs, and finally, the structure with the minimum free energy is chosen. The current update enhances Knotify+’s ability to recognize and predict even more complex motifs, while it maintains the same level of complexity. In practice, the additional computations increase the execution time of the algorithm but are slight enough to be considered acceptable.

## 2. Related Work

Most of the algorithms have encapsulated dynamic programming in their pipeline process in order to determine the most likely secondary structure of an RNA, trying to minimize the free energy [16,17]. Other approaches that have focused on pseudoknot prediction, e.g., [18], have enforced entropy, stability, and minimum free energy. The proof that this problem is NP (nondeterministic polynomial time)-complete [19] has encouraged the development of stochastic and heuristic methods [20,21,22]. A typical example is Knotty [23], which predicts pseudoknots, with a CCJ (Chen–Condon–Jabbari) algorithm [24] with sparsification. Additionally, ProbKnot [25] computes base pair probabilities of non-pseudoknotted substructures, building the secondary structure based on the maximum expected accuracy. IPknot [26] also leverages the advantages of integer programming and base pair probabilities, performing better than the previous methods. Its extension [27] calculates secondary structures with pseudoknots in linear time using the LinearPartition model and pseudo-expected accuracy. This improved version can handle long sequences in a reasonable execution time, but there is still room for improvement in terms of accuracy.

Other approaches such as Pfold [28,29], PPfold [30], and RNA-Decoder [31] predict the secondary structure by applying Stochastic Context-Free Grammar (SCFG). All these approaches are specialized in pattern recognition, so they reveal similarities in structures, and in turn, they can be fine-tuned by assigning appropriate weights to the rules. Other typical SCFG-based frameworks are Contrafold [32], Evfold [33], Infernal [34], and Oxfold [35]. The extensive research on SCFG-based methods reveals the need for the efficient collaboration of grammar and computation methods, heuristic and probabilistic algorithms, minimum free energy computation, maximum base pairing, base pairing probabilities, and other algorithmic and biological concepts. Therefore, it is crucial to find the optimal match between these concepts, to succeed in predicting the RNA secondary structure. In this direction, we propose a grammar-based framework, which leverages maximum base pairing and minimum free energy, creating an efficient prediction pipeline. However, the underlying model of the proposed methodology is that of Context-Free Grammar (CFG).

Machine learning algorithms have also been proposed in the literature. They endeavor to unveil hidden patterns by applying supervised and unsupervised methods in training datasets. The majority of these need large datasets because they use deep learning techniques which require a significant amount of data for the training process to avoid overfitting. In [36], for example, the authors used deep learning and tertiary constraints to tackle this task, while others, e.g., [8], have constructed bidirectional-LSTM (long short-term memory) networks and the IBPMP (improved base-pair maximization principle) to select the correct base pairs to then predict the optimal structure. Similarly, 2dRNA [37] applies a coupled two-staged deep neural network that provides data to a U-net architecture. A bidirectional LSTM encodes the data in a higher dimension, and at the final stage, a fully connected network decodes them, producing the dot-bracket structure. To predict the secondary structure, including pseudoknots, ATTfold [38] also adopts deep learning models by incorporating an attention mechanism as an encoder. It encodes a base pairing score matrix; then, a CNN (Convolutional Neural Network) decodes the data in an appropriate format. The training process takes place according to hard biological concepts, aiming to reduce structures that do not exist in nature in agreement with the folding rules.

## 3. Theoretical Background

Next, we provide background information about the core theoretical concepts such as RNA, pseudoknots, bulges and internal loops, and parsers. This information is necessary for the illustration of the proposed methodology in Section 4.

### 3.1. RNA

RNA is a single-stranded molecule that folds, forming a specific set of RNA base pairs, the Watson–Crick base pairs (A–U and G–C), [39] and, less frequently, the G–U wobble-base pair. Its secondary structure is a dominant component for the explanation of various biological processes. The nitrogenous bases A, C, G, U, sugars, and a phosphate backbone are combined to form RNA and its forms such as mRNA, tRNA, and rRNA, which are all involved in the production of protein; one of these, the mRNA, carries the genetic information. The most significant motifs and the most frequent in nature are those of loops, kissing loops, bulges, hairpins, and pseudoknots. Our contribution is a methodology that is concentrated on H-type pseudoknots, incorporating bulges and internal loops.

#### 3.1.1. The Pseudoknot Motif

**Figure 1:**
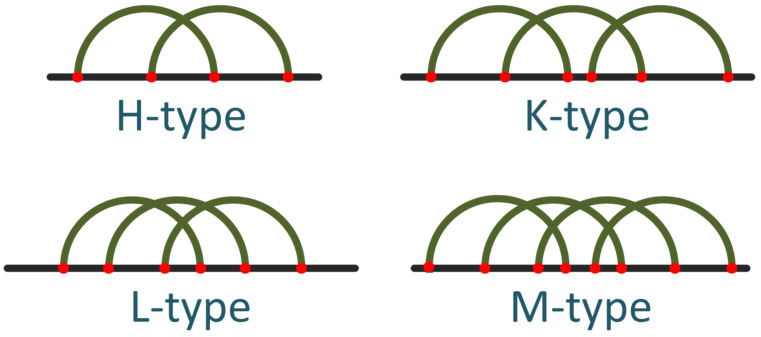
The most common types of pseudoknots (after [42]).

One of the least frequent patterns in RNA sequences, but challenging in terms of prediction, is the pseudoknot. A pseudoknot is said to exist when two base pairs intersect. This motif was initially observed in the Turnip Yellow Mosaic virus [40]. The simplest type of pseudoknot is formed by two single-stranded sections. Numerous variations have been observed, but the four main types [41] are the H, K, L, and M types, as shown in Figure 1 [42]. Specifically, the H-type pseudoknot [9] consists of two stems and two loops of arbitrary length. The intersection of a couple of base pairs (or core stems in our notation) leads to its creation.

#### 3.1.2. Bulges and Internal Loops

**Figure 2:**
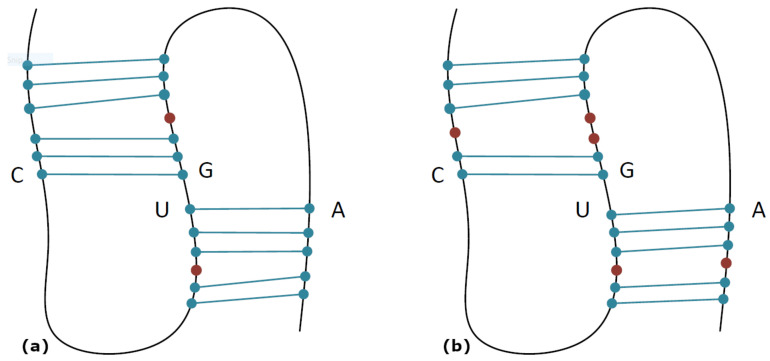
H-type pseudoknots with bulges (**a**) and internal loops (**b**). Unpaired bases forming a bulge or internal loop are represented with red dots.

A bulge is constructed by unpaired bases (A, U, G, and C) and its size may be from one to many unpaired bases. Their appearance in all types of structured functional RNAs [12] emphasizes their utmost importance and led our research to embody them in our pseudoknot prediction framework. To illustrate this motif, we present the unpaired bases that form a bulge with red dots in Figure 2a. Internal loops, which are also known as interior loops, may be created in an RNA sequence when the double-stranded RNA separates as a consequence of no pairing between the nucleotides. The difference between interior loops and stem loops is that interior loops exist in the middle of a stretch of double-stranded RNA. To illustrate this motif, we present the unpaired bases that form an internal loop with red dots in Figure 2b.

### 3.2. Syntactic Pattern Recognition

The proposed framework, Knotify+, is an extension of the work presented in [15], including prediction of bulges and internal loops. The underlying model in predicting pseudoknots of type H in [15] is that of context-free grammar (CFG). According to syntactic pattern recognition theory, a language [43], which is a collection of syntactic rules, should be initially defined. These rules construct parse trees that contain the string of interest at the terminal nodes. The grammar is comprised of a collection of syntax rules enriched with a vocabulary. According to them, we recognize the inclusion of a string of symbols in a specific language. As Noam Chomsky [44] proposed, grammar can be classified into four categories, known as the Chomsky hierarchy. Knotify+ encapsulates a CFG widely used in a considerable number of applications, such as speech processing and compilers [45].

#### Context-Free Grammar

In order to construct a CFG [46], four sets 〈NT,T,R, and S〉 should be defined. *S* is the start nonterminal symbol, terminal symbols and nonterminal symbols form the sets *T* and *NT*, respectively, and the syntactic rules form the set *R*. The notation of the syntax rules is L→δ, where L∈NT and $\delta \in {\left(T\cup NT\right)}^{*}$, defining that *L* is capable of producing a string of symbols δ.

Due to their high expressiveness, there is a considerable number of parsers in the literature. The most cited and widely used algorithms are the CYK [47] introduced by Cocke, Younger, and Kasami and the Earley parser [48]. Modifications of the abovementioned parsers are presented in [49,50,51] and as parallel versions in [52,53].

Knotify+, similarly to [15], encapsulates Yet Another Early Parser (YAEP) [54], which is a performant Earley’s parser implementation for ambiguous grammar and appropriate for our RNA pseudoknot prediction grammar.

## 4. Proposed Methodology

**Figure 3:**
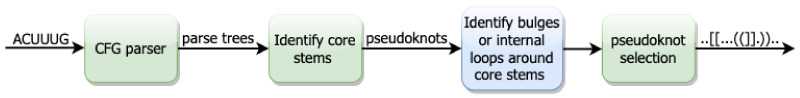
Overview of the Knotify+ proposed methodology.

In this section, the methodology proposed by the Knotify+ platform is presented. Knotify+ is an extension of the Knotify platform presented in [15], including the pruning technique presented in [42], capable of predicting bulges and internal loops around the core stems of the pseudoknot. Knotify manages to predict a pseudoknot in an RNA sequence making use of three main tasks: (a) a CFG parser analyzes the RNA sequence so that all trees in which a pseudoknot pattern is detected are generated; (b) the produced trees are parsed to detect the core stems that form the pseudoknot and the possible base pairs around the core stems of the pseudoknot, (c) the optimal tree is selected using two well-known criteria, that of the maximum number of base pairs and the minimum free energy of the sequence. A thorough analysis of the abovementioned tasks (see Figure 3) is provided in the next subsections. Knotify+ adds a new task (see the blue box in Figure 3) before the selection of the pseudoknot, which is responsible for the identification of bulges or internal loops around the core stems.

Consequently, the proposed implementation receives as input a string representing an RNA sequence of nitrogenous bases and produces the base pairing of the given RNA sequence in extended dot-bracket notation. The Knotify+ source code and implementation details are available as a public repository on GitHub [55].

### 4.1. CFG to Identify Pseudoknots

Knotify+’s methodology is based on the platform proposed in [15]. Hence, Knotify+ makes use of an efficient CFG parser. Therefore, initially, the appropriate primitive patterns should be selected. With regard to the RNA sequence representation, the obvious choice was to assign the nitrogenous bases A, C, G, and U to the characters “A”, “C”, “G”, and “U”, respectively, which also formed the set T of the terminal symbols of the grammar. The sequences of those four characters, such as AAUCCGG or CCGAAAUACG, formed a string that represents an RNA. After the primitive patterns were selected, a convenient grammar was defined, so as to syntactically analyze the linguistic representation of the original patterns.

The proposed platform makes use of the CFG $G_{RNA}$ that was initially presented and extensively described in [15]. Knotify+ initially executed the space elimination proposed in [42] aiming to dramatically decrease the substrings to be parsed by our sliding-windows technique. Then, the CGF parser analyzed the RNA sequence so that all trees in which a pseudoknot pattern was detected were generated. The main contribution of this paper is the creation of a new module that predicts bulges and internal loops around the core stems when the pseudoknot is decorated. This process is presented in Section 4.2. Finally, the last task of pseudoknot selection is executed as described in [42] and presented in Section 4.3.

### 4.2. Decorate Core Stems

During the first task, all parse trees were constructed by the parser. By the use of these trees, all possible pseudoknots and their core stems were allocated. The second task dealt with the traversing of all these trees to locate further stems. The CFG proposed in [15] was dedicated to detecting the initially crossing stems of the pseudoknot, in our notation the core stems, trying to amend the CFG parser’s efficiency. Consequently, all parse trees were evaluated for the possible detection of base pairs surrounding the pseudoknot’s core stems. All bases located in each of the two loops were consecutively checked for their ability to create a pair with a base in an appropriate position.

In Table 1, the process of the core stems decoration is presented. After the parser detected the core stems U–A and C–G at positions 10–17 and 5–11, the two pseudoknot loops were specified. The left loop was at positions 6 to 9, and the right loop was at positions 12 to 16. The bases in these loops were initially examined for whether they might pair with bases outside the pseudoknot’s loops. The base pairs in the left loop were tested for a match with bases at positions 18 to 22, while bases in the right were tested for a match with bases at positions 18 to 22.

In both loops of the pseudoknot, the base pairs at positions 9–18, 8–19, 4–12, and 3–14 were sequentially detected during stages 1 to 4, respectively. Table 1 presents this procedure in detail. Once no more sequential base pairs could be formed, the existence of bulges and internal loops was checked (stage 5). For each side, left or right, the unpaired bases were examined for whether they could form a base pair after creating a bulge or an internal loop. In our example, on the left side, the set at positions 6–7 may form base pairs with a set at positions 20–22 after creating bulges; those two sets were called the left pair of sets. On the right side, the set at positions 1–2 may form base pairs with a set at positions 14–16 after creating bulges; those two sets were called the right pair of sets. Users may define the maximum bulge size, which is given as an argument when the program is executed. This parameter is called the *maximum_bulge_size*. For each pair of sets, there may be a bulge of length 0 to *maximum_bulge_size* at one set and 0 to *maximum_bulge_size* at the other set. In the case where the bulge’s length is zero on one side and greater than zero on the other side, then a bulge is located. Otherwise, if the bulge’s length is greater than zero on both sides, then an internal loop is located. The Cartesian product of those cases was executed, and multiple dot-brackets strings were produced. By applying the criteria of the minimum free energy and the greatest number of base pairs of the pseudoknot, the optimal case was selected. The result of this procedure is shown in stage 5 of Table 1. Regarding the left pair of sets, there may be a base pair at positions 7–21 after creating a bulge at position 20. Regarding the right pair of sets, there may be a base pair at positions 2–16 after creating a bulge at positions 14–15, there may be a base pair at positions 1–14 after creating a bulge at position 2, or there may be a base pair at positions 1–15 after creating a bulge at position 2 and another one at position 14, creating in this way an internal loop. The last case was the one that was finally selected, as shown in stage 5, where the internal loop is highlighted in red.

Knotify+ allows the user to choose the option of the base pairs, U–G, as an argument from the command line, as well as the value of the *maximum_bulge_size*.

### 4.3. Optimal Tree Selection

Knotify+ incorporated a hybrid model to choose the optimal tree among the trees that were produced from the CFG. This task facilitated the maximum base pairing and the MFE (Minimum Free Energy) principles. In the first stage, it ranked all the produced trees according to the count of base pairs around the pseudoknot, excluding the stems formed after the bulges or internal loops. The next stage consisted of the application of the MFE in the trees that were ranked at the top in the first stage, i.e., the trees with the most base pairs around the pseudoknot. After extensive experiments, we observed that including all the possible detected stems after the bulges or internal loops may lead to excluding the correct RNA sequence from the top-ranking sequences (regarding base pairs count) that were promoted to the second stage of selection, that of the MFE calculation. Consequently, the first task of the proposed tree selection, that of maximum pairing, was applying it to the RNA sequences without taking into consideration the stems detected after the bulges or internal loops.

**Figure 4:**
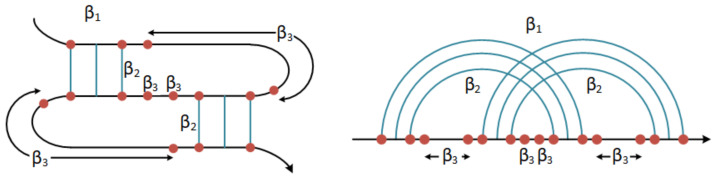
$\beta_{1}$ is the weight or cost of the existence of a pseudoknot; $\beta_{2}$ is the cost of the core stems; $\beta_{3}$ is the cost of the unpaired bases inside the pseudoknot (after [56]).

Finally, the secondary structure with the minimum free energy was selected. A module derived from HotKnots [56] calculated each candidate’s energy and, in turn, provided the energy scores to Knotify+ to make the final selection. The energy calculation algorithm was introduced by Mathews [57], but we used a variation based on [58] presented in the following relation:

(1) $$G^{pseudo}=\beta_{1}+\beta_{2}*B^{p}+\beta_{3}*U^{p},$$

where $\beta_{1}$ is the weight or cost of the existence of a pseudoknot; $B^{p}$ is the total number of core stems; $U^{p}$ is the total number of unpaired bases inside the pseudoknot. Following the experimental evaluation in [56], we set the parameters $\beta_{2}$ (cost for the core stems) and $\beta_{3}$ (cost for the unpaired bases) equal to 0.1 and $\beta_{1}$ equal to 9.6 (see Figure 4).

## 5. Performance Evaluation

### 5.1. Dataset Construction

To evaluate Knotify+’s accuracy against other methodologies, a dataset [59] consisting of 260 well-known RNA sequences including pseudoknots, was constructed. A considerable number of these sequences formed bulges or internal loops after their core stems of the pseudoknot. The dataset was separated into four sets by length. The first set consisted of 75 RNA sequences of lengths smaller than 30, the second had 67 RNA sequences of lengths between 30 and 40, the third had 55 RNA sequences of lengths between 40 and 50, and the last set had 63 RNA sequences of lengths greater than or equal to 50. The sequences were selected from the RNA Database platforms [60,61] that provide publicly available data. The proposed methodology was compared against two efficient implementations proposed in the literature, i.e., IPknot and Knotty [23,26], as well as the previous version of our implementation. Consequently, four platforms were used during the performance evaluation task, i.e., IPknot, Knotty, Knotify, and Knotify+.

### 5.2. Methods of Evaluation

In measuring our framework’s performance, three methods were chosen: (a) the percentage of the pseudoknot’s core stems prediction, (b) the confusion matrix including the precision (PPV), recall, F1-score, and MCC (Matthews correlation coefficient), and (c) the execution time. Concerning the Knotify+ platform, all experiments were implemented with the parameter *maximum_bulge_size* equal to 3.

#### 5.2.1. Pseudoknots’ Core Stems Prediction

In Table 2, the capability of each platform of predicting the core stems of the pseudoknots is presented. The second column presents the number of pseudoknots for which a platform succeeded in predicting both core stems, while the fourth column presents the number of pseudoknots for which a platform succeeded in predicting just one core stem. The proposed methodology, Knotify+, similar to Knotify, detected both core stems of the pseudoknot perfectly in 142 out of 260 sequences, while IPknot did so in 38 sequences and Knotty in 121 sequences. Moreover, Knotify+, succeeded in additionally detecting one core stem of the pseudoknot in 45 sequences, while IPknot did so in 22 sequences, Knotty in 47 sequences, and Knotify in 38 sequences. Consequently, Knotify+ outperformed the other platforms, succeeding in predicting at least one core stem in 63.27% of the dataset’s sequences, with IPknot at 18.85%, Knotty at 55.58%, and Knotify at 61.92%. This finding demonstrates that even in cases where the exact prediction was not feasible, Knotify+ predicted at least one core stem better than our previous implementation Knotify and the other two well-known platforms.

**Figure 5:**
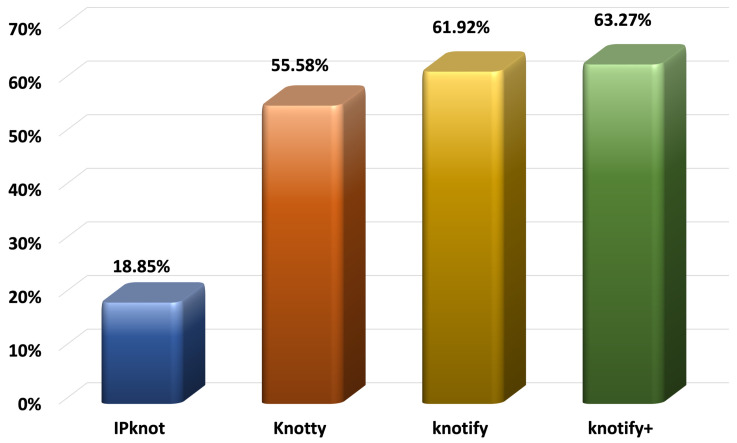
Percentage of at least one core stem prediction for each method.

Adopting the methodology of locating the pseudoknot proposed in [57], we permitted the location of the one base of each stem to be moved one position on the right or left. Consequently, pair (*k*, *l*) was equivalent to (*k* ± 1, *l*) or (*k*, *l*± 1). The results of predicting the pseudoknots’ core stems are also shown in Figure 5.

#### 5.2.2. Confusion Matrix, Precision, Recall, F1-Score, and MCC

The performance of all platforms regarding the precision, the recall, the F1-score, and the Matthews Correlation Coefficient (MCC) is presented in Table 3. The definitions of these metrics are presented in Equations (2)–(5). In Equations (2)–(5), *tp* (true positive) expresses the count of the correctly predicted base pairs, *fp* (false positive)—thecount of the incorrectly predicted base pairs, *fn* (false negative)—the count of the base pairs that were not predicted, and *tn* (true negative)—the count of those correctly not predicted.

(2) $$PPV=\frac{tp}{tp+fp}$$

(3) $$Recall=\frac{tp}{tp+fn}$$

(4) $$F1-score=\frac{2\times PPV\times Recall}{PPV+Recall}$$

(5) $$MCC=\frac{tp\times tn-fp\times fn}{\sqrt{\left(tp+fp)(tp+fn)(tn+fp)(tn+fn\right)}}$$

As shown in Table 3, the proposed methodology outperformed the previous version of Knotify regarding the recall, F1-score, and MCC and also reduced the distance from Knotty, which still had better performance at those metrics. In addition, regarding the precision metric, Knotify+ maintained better performance than Knotty, as Knotify did. Knotify+ achieved a greater number of tp than Knotify, a fact that showed the improvement in the prediction, but its attempt to add stems after bulges or interior loops increased the number of fp and therefore decreased the precision. Despite this reduction in precision, the F1-score, the harmonic mean of the precision and recall, and the metric that describes the prediction rate overall was higher in Knotify+ than in Knotify. Finally, IPknot had the lowest performance in all metrics.

In Table 4, the confusion matrices are presented and divided into four sets for each method, providing the *tp*, *tn*, *fp*, and *fn* in detail. Knotify+ counted more tp and lower or equal fp and fn for the sequences smaller than 40 (L < 30 and 30 ≤ L < 40) compared to the evaluated methods. Its prediction capability in sequences larger than 40 (40 ≤ L < 50 and L ≥ 50) was better than Knotify’s and comparable to but still lower than Knotty’s, which increased its prediction capability when the length increased.

**Figure 6:**
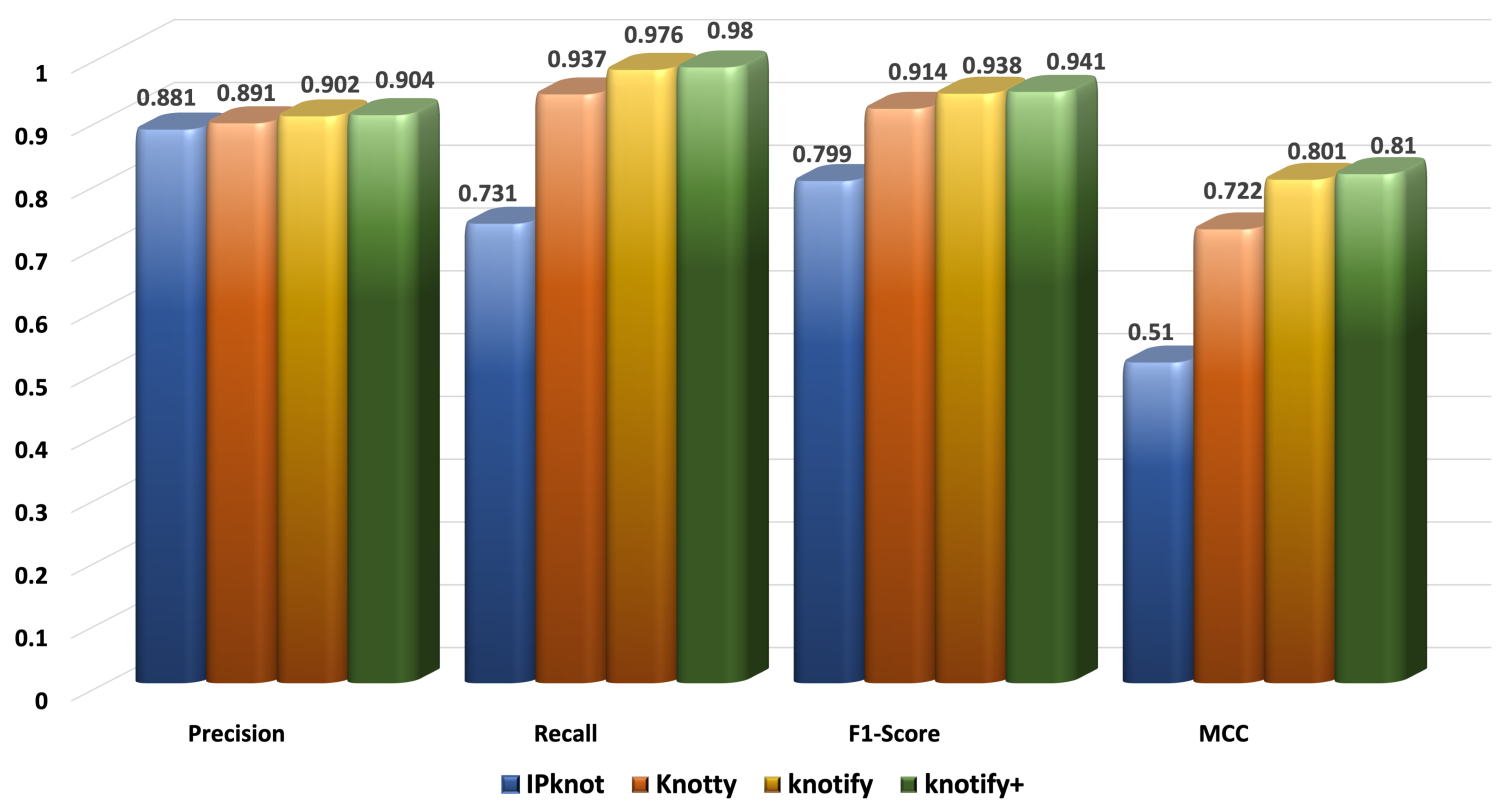
Metrics for the sequences of length < 30.

**Figure 7:**
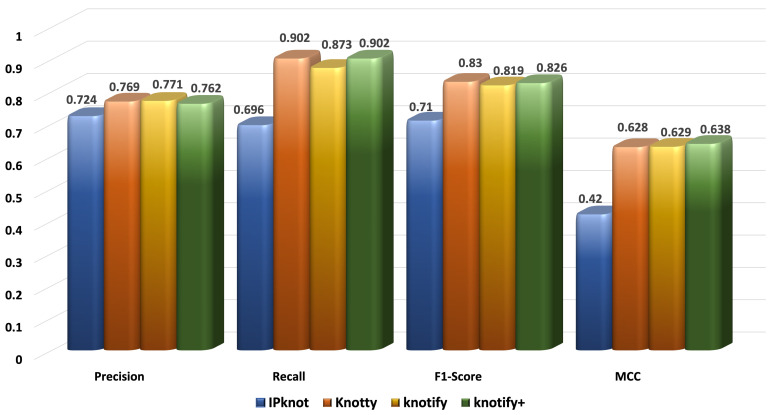
Metrics for the sequences of length ≥ 30 and < 40.

**Figure 8:**
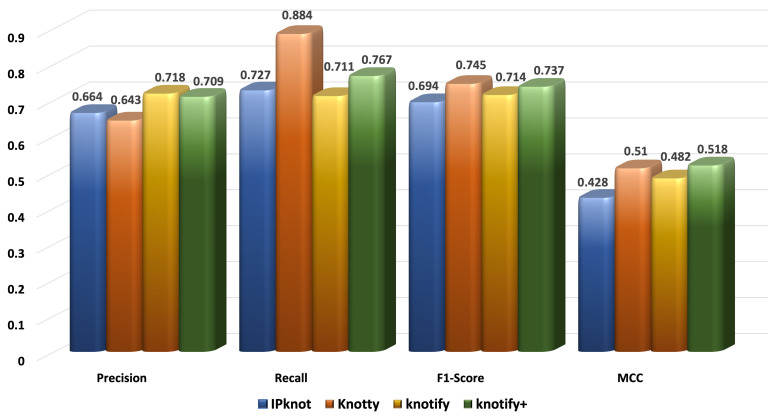
Metrics for the sequences of length ≥ 40 and < 50.

**Figure 9:**
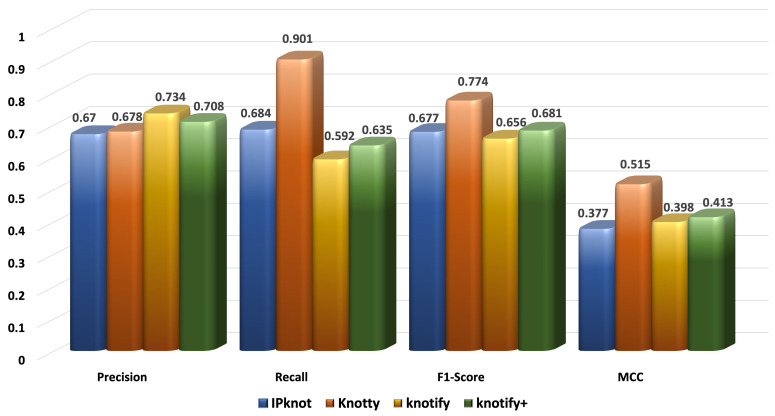
Metrics for the sequences of length ≥ 50.

Figure 6, Figure 7, Figure 8 and Figure 9 present the results for each metric per set depending on the length. In evaluating these figures, our methodology typically outperformed all the methods in all the metrics when the length was smaller than 30. In the sequences between 30 and 40, Knotify+ was still more efficient according to the F1-score and the MCC because of its high recall and comparable precision rate. In the sequences between 40 and 50, Knotify+ outperformed Knotify in all metrics and was equivalent to Knotty regarding the F1-score and MCC. Finally, for the sequences larger than 50, Knotty outperformed the other methodologies. The main reason for this superiority is that as the sequence’s length increased, there were more motifs apart from pseudoknots, bulges, and internal loops, for example, hairpins, which Knotify+ was inherently not capable of predicting in this version. These structures may be located by Knotty, augmenting its tp score, leading to higher recall and F1-score metrics. Having observed this fact, our research team has set, as a future goal, adding to the platform the ability to detect additional complex patterns in the loops of the pseudoknot.

#### 5.2.3. Execution-Time Comparison

The third metric was the execution time, where the proposed methodology was tested against the other platforms in terms of time efficiency. Table 5 illustrates the required prediction time for each method.

The second column of Table 5 presents the required per platform execution time for the whole dataset. Knotify+ required 74.05 s; IPknot required 117.02 s; Knotty required 582.91 s. Obviously, Knotify+ was approximately eight times (582.9/74.05 = 7.87) faster than Knotty. The third column presents the average execution time for each method.

## 6. Conclusions

The prediction of the RNA secondary structure is quite a challenging task, especially for pseudoknotted structures. In this context, we proposed an intelligent grammar-based algorithm that predicted H-type pseudoknots with bulges and internal loops. It detected the secondary structure performant, and its accuracy was comparable to well-known platforms. Especially for sequences smaller than 30 bases, it outperformed all the examined methods, showing that the enhancement of its expressiveness led to an important advancement of our previous version. The most notable finding was that the proposed methodology outperformed our previous version Knotify regarding the recall, F1-score, and MCC in all sets, showing a significant improvement for sequences larger than 40. In addition, Knotify+ continued to outperform Knotty for small sequences, while it was comparable for sequences between 30 and 50, and significantly decreased the gap with Knotty for sequences larger than 50 bases. Meanwhile, Knotify+ maintained the highest percentage of core stems prediction compared to all the examined methods and was approximately eight times faster than Knotty.

## Figures and Tables

**Table 1:** The decoration process around the core stems of an H-type pseudoknot. Unpaired bases forming a bulge or internal loop are represented with red dots.

Position	1	2	3	4	5	6	7	8	9	10	11	12	13	14	15	16	17	18	19	20	21	22
**String**	A	C	A	U	C	C	G	C	C	U	G	A	U	U	U	G	A	G	C	A	C	A
**Core stems**:	.	.	.	.	[	.	.	.	.	(	]	.	.	.	.	.	)	.	.	.	.	.
**Stage 1**	.	.	.	.	[	.	.	.	(	(	]	.	.	.	.	.	)	)	.	.	.	.
**Stage 2**	.	.	.	.	[	.	.	(	(	(	]	.	.	.	.	.	)	)	)	.	.	.
**Stage 3**	.	.	.	[	[	.	.	(	(	(	]	]	.	.	.	.	)	)	)	.	.	.
**Stage 4**	.	.	[	[	[	.	.	(	(	(	]	]	]	.	.	.	)	)	)	.	.	.
**Stage 5**	[	.	[	[	[	.	(	(	(	(	]	]	]	.	]	.	)	)	)	.	)	.

**Table 2:** Pseudoknot location prediction according to the core stems in the whole dataset.

Platform	2 Matches	2 Matches (%)	1 Match	At Least 1 Match (%)
IPknot	38	14.62	22	18.85
Knotty	121	46.54	47	55.58
Knotify	142	54.62	38	61.92
Knotify+	142	54.62	45	63.27

**Table 3:** The confusion matrix for each method in the entire dataset.

Platform	*tp*	*tn*	*fp*	*fn*	Precision	Recall	F1-Score	MCC
IPknot	3850	3746	1488	1606	0.721	0.706	0.713	0.421
Knotty	5006	3331	1836	517	0.732	0.906	0.810	0.574
Knotify	4170	4061	1154	1305	0.783	0.762	0.772	0.540
Knotify+	4342	3975	1306	1053	0.769	0.805	0.786	0.558

**Table 4:** The confusion matrices for each method per set.

Length	L < 30					30 ≤ L < 40				40 ≤ L < 50				L ≥ 50			
**Platform**		***tp***	***tn***	***fp***	***fn***	***tp***	***tn***	***fp***	***fn***	***tp***	***tn***	***fp***	***fn***	***tp***	***tn***	***fp***	***fn***
**IPknot**		916	514	124	337	824	810	294	355	754	897	396	284	1368	1519	674	631
**Knotty**		1196	469	146	80	1064	786	316	117	894	803	510	124	1848	1264	876	204
**Knotify**		1230	490	132	39	748	991	288	304	748	991	288	304	1218	1723	420	831
**Knotify+**		1248	486	132	25	1010	847	316	110	798	1004	328	242	1286	1638	530	676

**Table 5:** The execution time required for each method in the whole dataset.

Platform	Total Time (s)	Average Time (s)
IPknot	117.02	0.45
Knotty	582.91	2.24
Knotify	56.43	0.22
Knotify+	74.05	0.28

## Data Availability

Not applicable.

## Abbreviations

The following abbreviations are used in this manuscript:

AI	Artificial Intelligence
CFG	Context-free grammar
CCJ	Chen–Condon–Jabbari
CNN	Convolutional Neural Network
CYK	Cocke–Younger–Kasami
DNA	Deoxyribonucleic acid
IBPMP	Improved base-pair maximization principle
LSTM	Long short-term memory
MCC	Matthews correlation coefficient
MFE	Minimum free energy
NP	Nondeterministic polynomial
RNA	Ribonucleic acid
SCFG	Stochastic context-free grammar
YAEP	Yet another early parser

## References

[1] Crick F. (1970). Central Dogma of Molecular Biology. Nature.

[2] Wu L., Belasco J. (2008). Let Me Count the Ways: Mechanisms of Gene Regulation by miRNAs and siRNAs. Mol. Cell.

[3] Rossi J. (2004). Ribozyme diagnostics comes of age. Chem. Biol..

[4] Shi Y. (2014). A Glimpse of Structural Biology through X-ray Crystallography. Cell.

[5] Barnwal R., Yang F., Varani G. (2017). Applications of NMR to structure determination of RNAs large and small. Arch. Biochem. Biophys..

[6] Zuker M. (2000). Calculating nucleic acid secondary structure. Curr. Opin. Struct. Biol..

[7] Nussinov R., Jacobson A.B. (1980). Fast algorithm for predicting the secondary structure of single-stranded RNA. Proc. Natl. Acad. Sci. USA.

[8] Wang L., Liu Y., Zhong X., Liu H., Lu C., Li C., Zhang H. (2019). DMfold: A novel method to predict RNA secondary structure with pseudoknots based on deep learning and improved base pair Maximization Principle. Front. Genet..

[9] Staple D.W., Butcher S.E. (2005). Pseudoknots: RNA structures with diverse functions. PLoS Biol..

[10] Wyatt J., Puglisi J., Tinoco I. (1989). RNA folding: Pseudoknots, loops and bulges. Bioessays.

[11] Turner D. (1992). Bulges in nucleic acids. Curr. Opin. Struct. Biol..

[12] Hermann T., Patel D. (2000). RNA bulges as architectural and recognition motifs. Structure.

[13] Wu H., Uhlenbeck O. (1987). Role of a bulged A residue in a specific RNA-protein interaction. Biochemistry.

[14] Woese C., Gutell R. (1989). Evidence for several higher order structural elements in ribosomal RNA. Proc. Natl. Acad. Sci. USA.

[15] Andrikos C., Makris E., Kolaitis A., Rassias G., Pavlatos C., Tsanakas P. (2022). Knotify: An Efficient Parallel Platform for RNA Pseudoknot Prediction Using Syntactic Pattern Recognition. Methods Protoc..

[16] Lorenz R., Bernhart S., Höner zu Siederdissen C., Tafer H., Flamm C., Stadler P., Hofacker I. (2011). ViennaRNA package 2.0. Algorithms Mol. Biol..

[17] Zuker M. (2003). Mfold web server for nucleic acid folding and hybridization prediction. Nucleic Acids Res..

[18] Cao S., Chen S. (2009). Predicting structures and stabilities for H-type pseudoknots with interhelix loops. RNA.

[19] Akutsu T. (2000). Dynamic programming algorithms for RNA secondary structure prediction with pseudoknots. Discret. Appl. Math..

[20] Meyer I.M., Miklos I. (2007). SimulFold: Simultaneously inferring RNA structures including pseudoknots, alignments, and trees using a Bayesian MCMC framework. PLoS Comput. Biol..

[21] Van Batenburg F., Gultyaev A.P., Pleij C.W. (1995). An APL-programmed genetic algorithm for the prediction of RNA secondary structure. J. Theor. Biol..

[22] Isambert H., Siggia E.D. (2000). Modeling RNA folding paths with pseudoknots: Application to hepatitis delta virus ribozyme. Proc. Natl. Acad. Sci. USA.

[23] Jabbari H., Wark I., Montemagno C., Will S. (2018). Knotty: Efficient and accurate prediction of complex RNA pseudoknot structures. Bioinformatics.

[24] Chen H.L., Condon A., Jabbari H. (2009). An O(n(5)) algorithm for MFE prediction of kissing hairpins and 4-chains in nucleic acids. J. Comput. Biol..

[25] Bellaousov S., Mathews D.H. (2010). ProbKnot: Fast prediction of RNA secondary structure including pseudoknots. RNA.

[26] Sato K., Kato Y., Hamada M., Akutsu T., Asai K. (2011). IPknot: Fast and accurate prediction of RNA secondary structures with pseudoknots using integer programming. Bioinformatics.

[27] Sato K., Kato Y. (2021). Prediction of RNA secondary structure including pseudoknots for long sequences. Brief. Bioinform..

[28] Knudsen B., Hein J. (1999). RNA secondary structure prediction using stochastic context-free grammars and evolutionary history. Bioinformatics.

[29] Knudsen B., Hein J. (2003). Pfold: RNA secondary structure prediction using stochastic context-free grammars. Nucleic Acids Res..

[30] Sukosd Z., Knudsen B., Vaerum M., Kjems J., Andersen E.S. (2011). Multithreaded comparative RNA secondary structure prediction using stochastic context-free grammars. BMC Bioinform..

[31] Pedersen J.S., Meyer I.M., Forsberg R., Simmonds P., Hein J. (2004). A comparative method for finding and folding RNA secondary structures within protein-coding regions. Nucleic Acids Res..

[32] Do C.B., Woods D.A., Batzoglou S. (2006). CONTRAfold: RNA secondary structure prediction without physics-based models. Bioinformatics.

[33] Pedersen J.S., Bejerano G., Siepel A., Rosenbloom K., Lindblad-Toh K., Lander E.S., Kent J., Miller W., Haussler D. (2006). Identification and classification of conserved RNA secondary structures in the human genome. PLoS Comput. Biol..

[34] Nawrocki E.P., Kolbe D.L., Eddy S.R. (2009). Infernal 1.0: Inference of RNA alignments. Bioinformatics.

[35] Anderson J.W., Haas P.A., Mathieson L.A., Volynkin V., Lyngsø R., Tataru P., Hein J. (2013). Oxfold: Kinetic folding of RNA using stochastic context-free grammars and evolutionary information. Bioinformatics.

[36] Singh J., Hanson J., Paliwal K., Zhou Y. (2019). RNA secondary structure prediction using an ensemble of two-dimensional deep neural networks and transfer learning. Nat. Commun..

[37] Kangkun M., Jun W., Yi X. (2020). Prediction of RNA secondary structure with pseudoknots using coupled deep neural networks. Biophys. Rep..

[38] Wang Y., Liu Y., Wang S., Liu Z., Gao Y., Zhang H., Dong L. (2020). ATTfold: RNA secondary structure prediction with pseudoknots based on attention mechanism. Front. Genet..

[39] Watson J., Crick F. (2003). Molecular Structure Of Nucleic Acids. Am. J. Psychiatry.

[40] Rietveld K., Van Poelgeest R., Pleij C.W., Van Boom J., Bosch L. (1982). The tRNA-Uke structure at the 3’ terminus of turnip yellow mosaic virus RNA. Differences and similarities with canonical tRNA. Nucleic Acids Res..

[41] Kucharík M., Hofacker I.L., Stadler P.F., Qin J. (2016). Pseudoknots in RNA folding landscapes. Bioinformatics.

[42] Makris E., Kolaitis A., Andrikos C., Moulos V., Tsanakas P., Pavlatos C. (2022). An intelligent grammar-based platform for RNA H-type pseudoknot prediction. Artificial Intelligence Applications and Innovations, Proceedings of the AIAI 2022 IFIP WG 12.5 International Workshops, IFIP Advances in Information and Communication Technology.

[43] Hopcroft J.E., Ullman J.D. (1969). Formal Languages and Their Relation to Automata.

[44] Chomsky N. (1956). Three models for the description of language. IRE Trans. Inf. Theory.

[45] Sipser M. (2006). Introduction to the Theory of Computation.

[46] Aho A.V., Lam M.S., Sethi R., Ullman J.D. (2006). Compilers: Principles, Techniques, and Tools.

[47] Younger D.H. (1967). Recognition and parsing of context-free languages in *n*^3^. Inf. Control..

[48] Earley J. (1970). An efficient context-free parsing algorithm. Commun. ACM.

[49] Graham S.L., Harrison M.A., Ruzzo W.L. (1980). An improved context-free recognizer. ACM Trans. Program. Lang. Syst..

[50] Ruzzo W.L. (1978). General Context-Free Language Recognition. Ph.D. Thesis.

[51] Geng T., Xu F., Mei H., Meng W., Chen Z., Lai C. (2014). A practical GLR parser generator for software reverse engineering. JNW.

[52] Pavlatos C., Dimopoulos A.C., Koulouris A., Andronikos T., Panagopoulos I., Papakonstantinou G. (2009). Efficient reconfigurable embedded parsers. Comput. Lang. Syst. Struct..

[53] Chiang Y., Fu K. (1984). Parallel parsing algorithms and VLSI implementations for syntactic pattern recognition. IEEE Trans. Pattern Anal. Mach. Intell..

[54] https://github.com/vnmakarov/yaep.

[55] https://github.com/ntua-dslab/Knotify/releases/tag/04-Knotify+.

[56] Ren J., Rastegari B., Condon A., Hoos H.H. (2005). HotKnots: ?Heuristic prediction of RNA secondary structures including pseudoknots. RNA.

[57] Mathews D., Sabina J., Zuker M., Turner D. (1999). Expanded sequence dependence of thermodynamic parameters improves prediction of RNA secondary structure1. J. Mol. Biol..

[58] Dirks R., Pierce N. (2003). Introduction A Partition Function Algorithm for Nucleic Acid Secondary Structure Including Pseudoknots. J. Comput. Chem..

[59] https://bit.ly/Knotify_plus_dataset_mdpi.

[60] Taufer M., Licon A., Araiza R., Mireles D., Van Batenburg F., Gultyaev A., Leung M. (2009). PseudoBase++: An extension of PseudoBase for easy searching, formatting and visualization of pseudoknots. Nucleic Acids Res..

[61] Danaee P., Rouches M., Wiley M., Deng D., Huang L., Hendrix D. (2018). bpRNA: Large-scale automated annotation and analysis of RNA secondary structure. Nucleic Acids Res..

